# Supplementary figures and images for: Postnatal maternal care moderates the effects of prenatal bisphenol exposure on offspring neurodevelopmental, behavioral, and transcriptomic outcomes
Source: PLoS One. 2024 Jun 11;19(6):e0305256. doi: 10.1371/journal.pone.0305256 (PMC11166292; doi:10.1371/journal.pone.0305256)

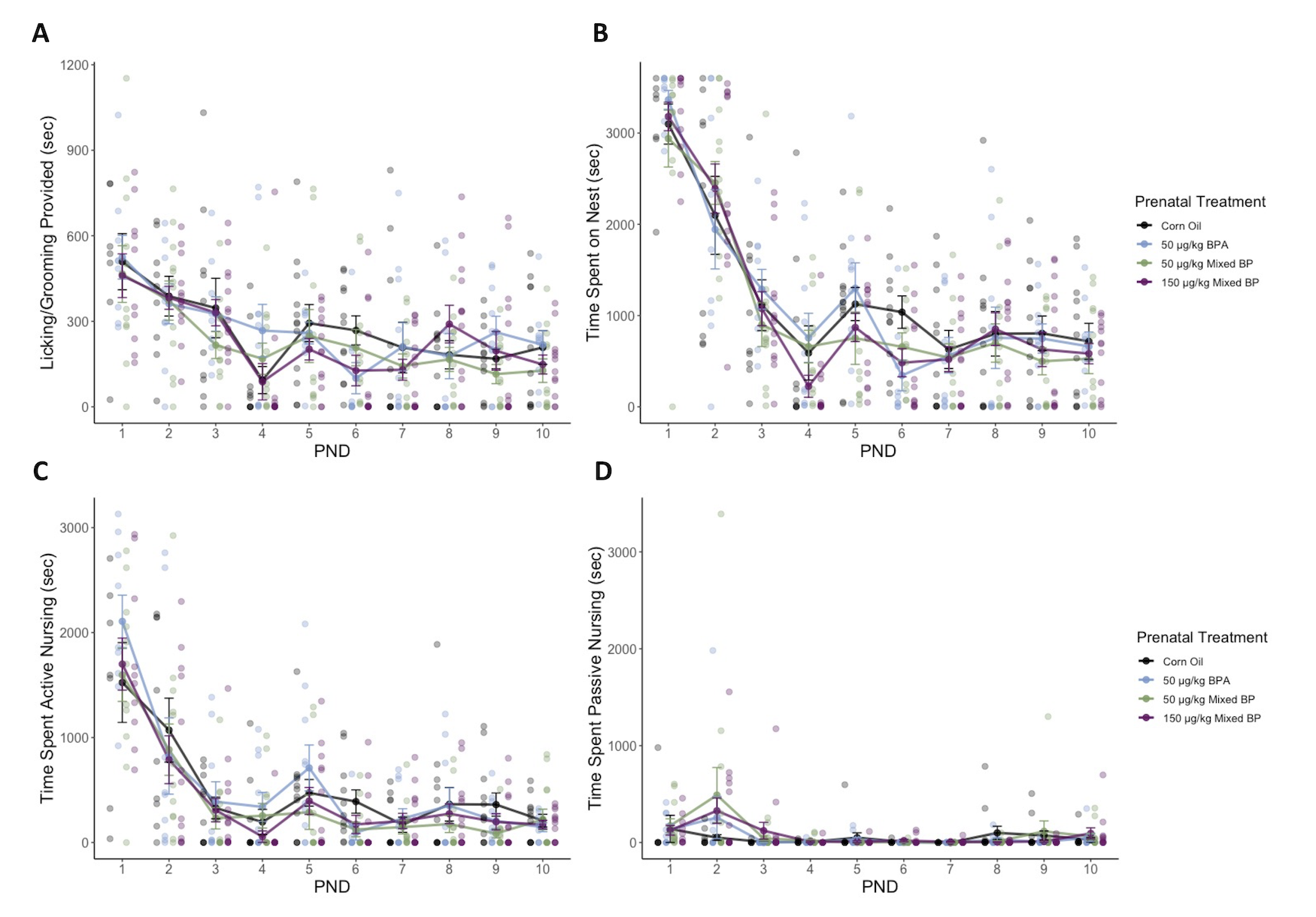

Supplement: S1 Fig — No main effects of prenatal treatment were found with (A) licking/grooming provisioning, (B) nest attendance, (C) active nursing, or (D) passive nursing. Line graphs are displayed with mean +/- SEM for each prenatal treatment group with individual datapoints. (TIFF) [file pone.0305256.s002.tiff]

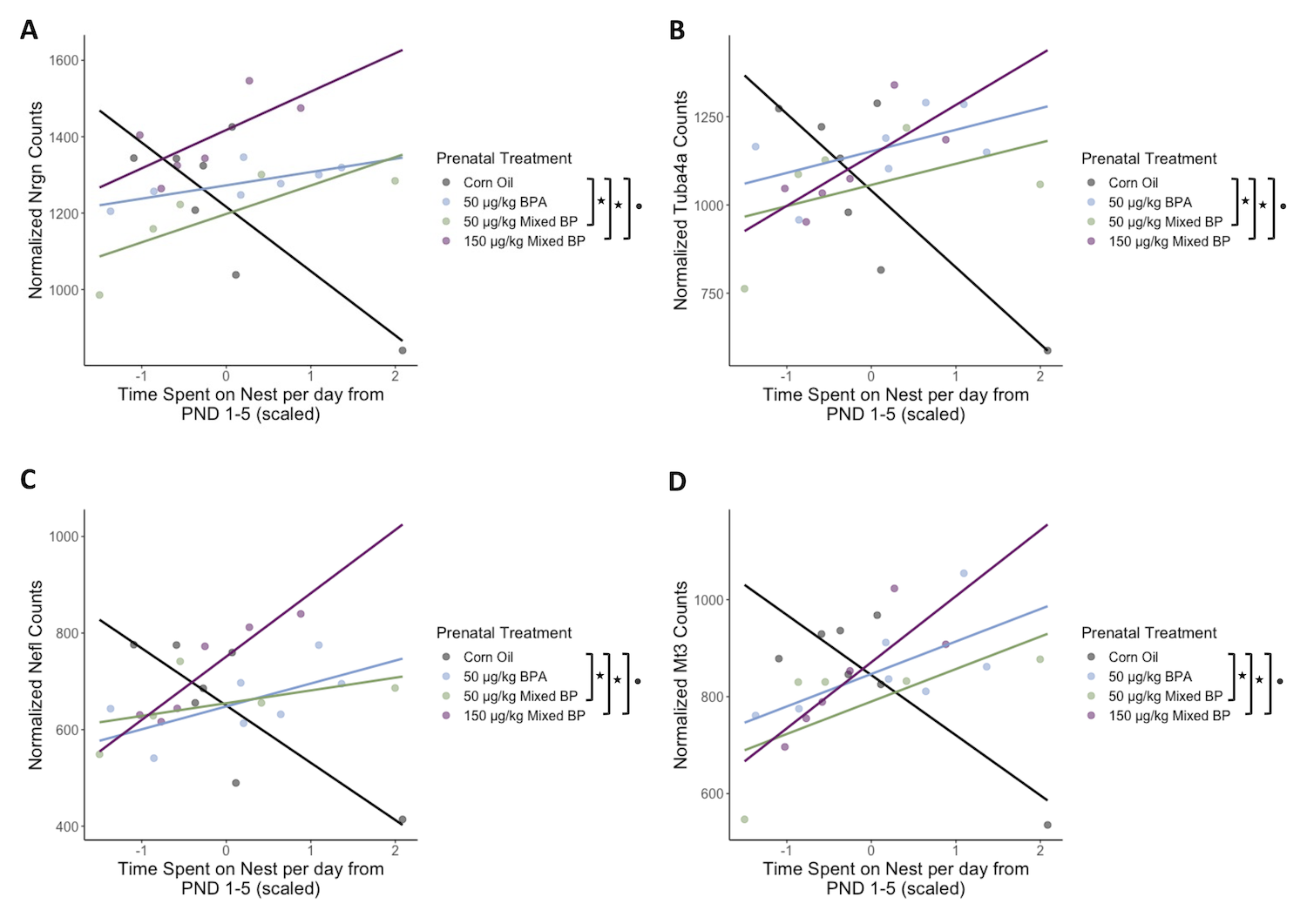

Supplement: S2 Fig — A significant main effect of postnatal nest attendance and significant interactions between prenatal treatment (50 μg/kg Mixed BP and 150 μg/kg Mixed BP) and postnatal nest attendance were found for (A) Nrgn, (B) Tuba4a, (C) Nefl, and (D) Mt3. Scatterplots are displayed with linear regression lines for each prenatal treatment group. ● p < 0.05 main effect of postnatal maternal care; + p < 0.10 main effect of postnatal maternal care; ★ p < 0.05 interaction between prenatal treatment and postnatal maternal care. (TIFF) [file pone.0305256.s003.tiff]

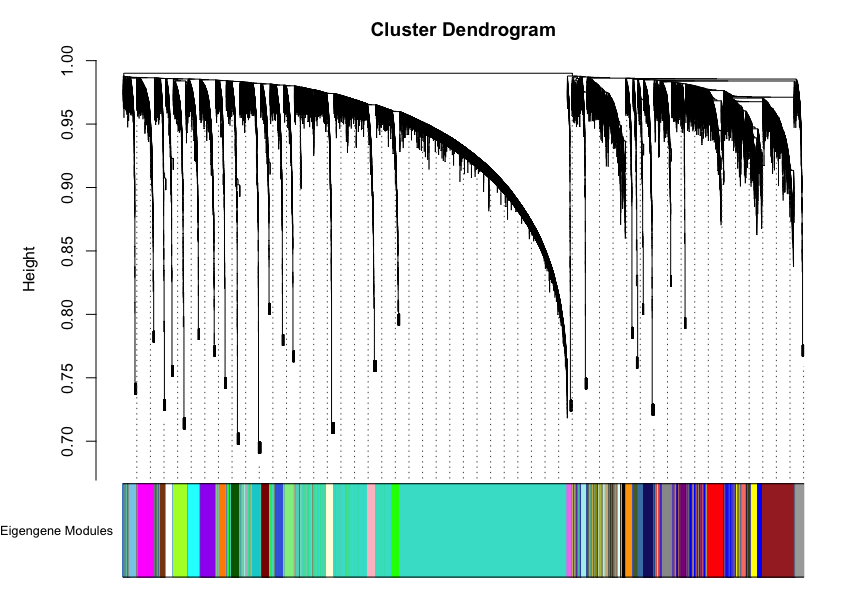

Supplement: S3 Fig — (TIFF) [file pone.0305256.s004.tiff]

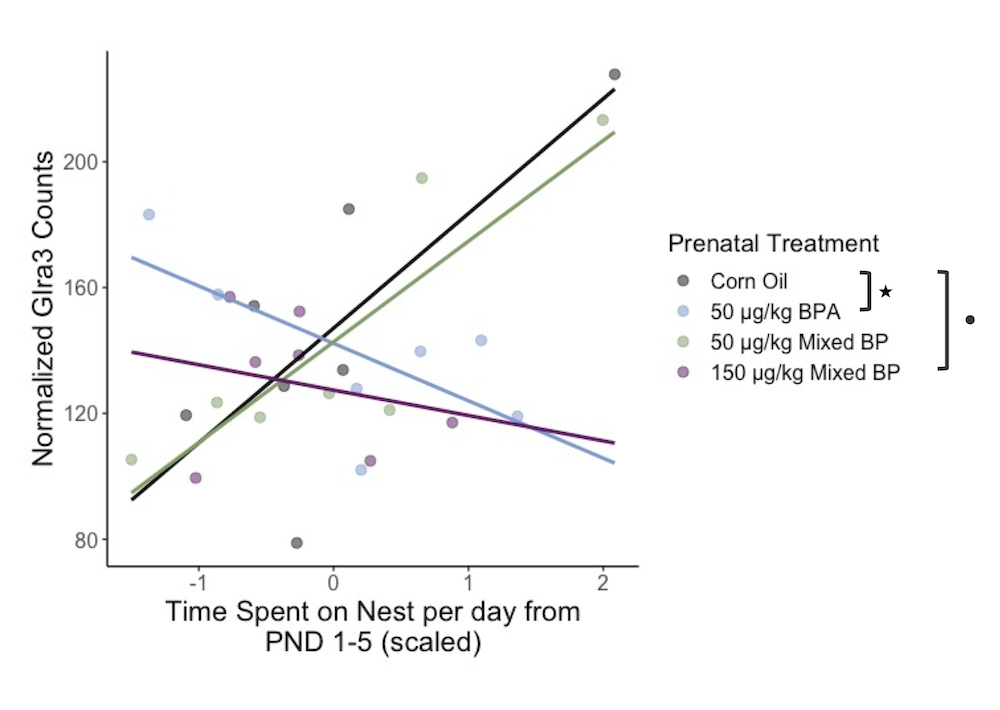

Supplement: S4 Fig — There was a significant main effect of postnatal nest attendance and significant interaction between prenatal treatment (50 μg/kg Mixed BP) and postnatal nest attendance. Scatterplot is displayed with linear regression lines for each prenatal treatment group. ● p < 0.05 main effect of postnatal maternal care; ★ p < 0.05 interaction between prenatal treatment and postnatal maternal care. (TIFF) [file pone.0305256.s005.tiff]

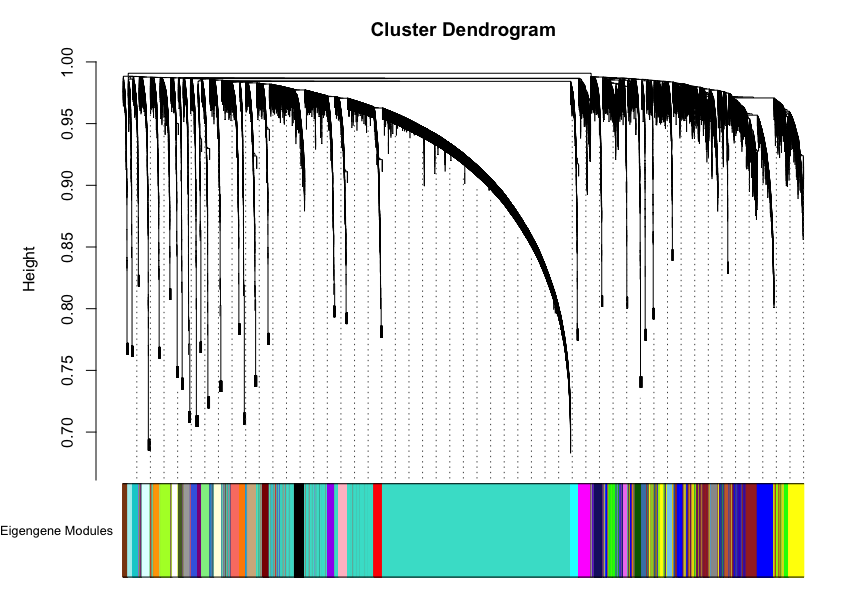

Supplement: S5 Fig — (TIFF) [file pone.0305256.s006.tiff]

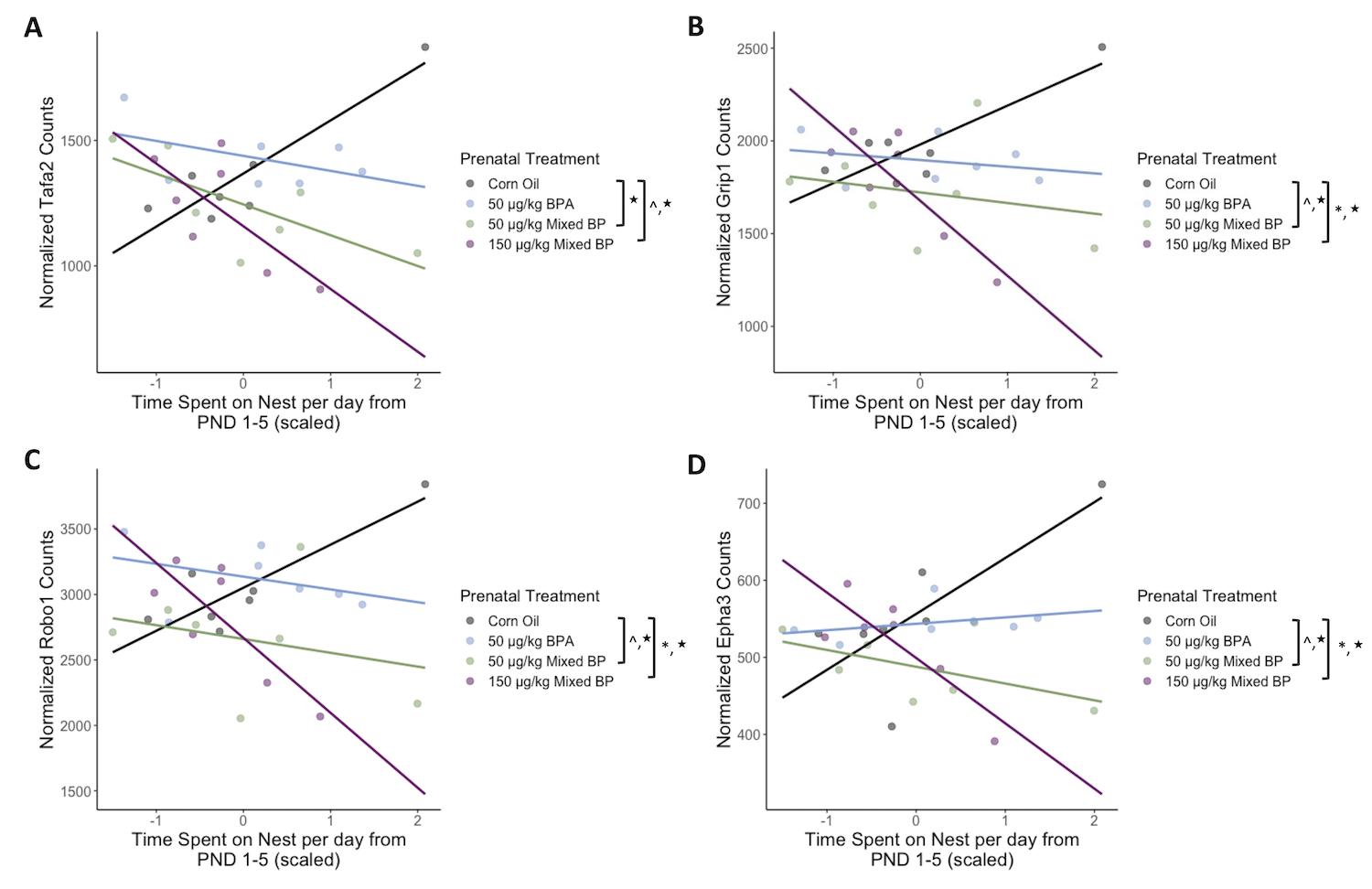

Supplement: S6 Fig — Significant or marginal main effects of prenatal treatment (50 μg/kg Mixed BP and 150 μg/kg Mixed BP) and significant interactions between prenatal treatment and postnatal nest attendance were found for (A) Tafa2, (B) Grip1, (C) Robo1, and (D) Epha3. Scatterplots are displayed with linear regression lines for each prenatal treatment group. ^ p < 0.10 main effect of prenatal treatment; * p < 0.05 main effect of prenatal treatment; ★ p < 0.05 interaction between prenatal treatment and postnatal maternal care. (TIFF) [file pone.0305256.s007.tiff]

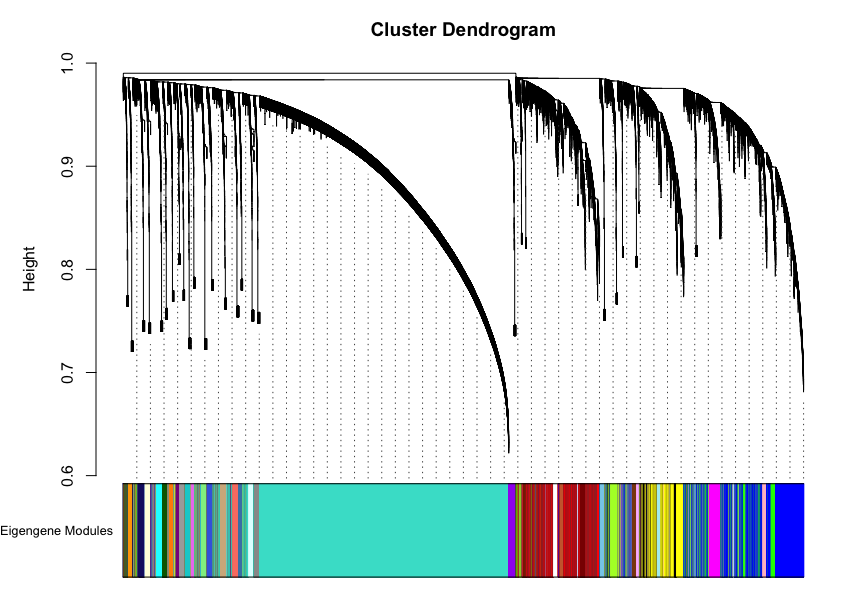

Supplement: S7 Fig — (TIFF) [file pone.0305256.s008.tiff]

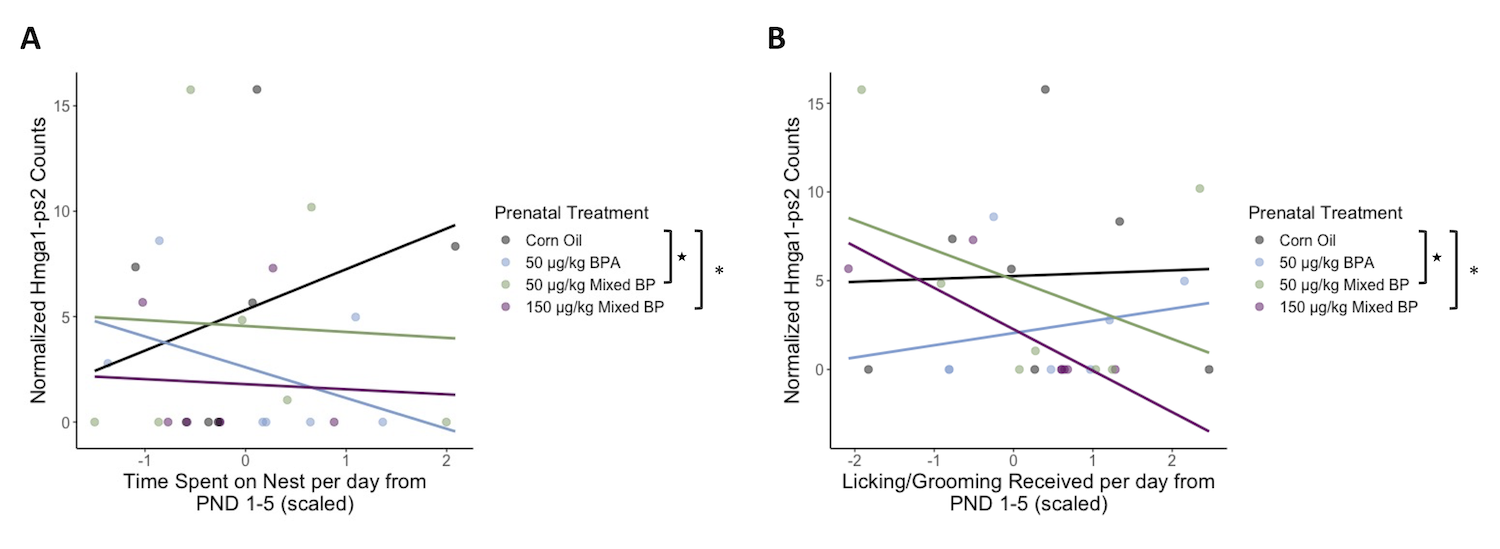

Supplement: S8 Fig — There was a significant main effect of prenatal treatment (150 μg/kg Mixed BP) and significant interactions between (A) prenatal treatment (50 μg/kg Mixed BP) and postnatal nest attendance and (B) prenatal treatment (50 μg/kg Mixed BP) and licking/grooming. Scatterplots are displayed with linear regression lines for each prenatal treatment group. * p < 0.05 main effect of prenatal treatment; ★ p < 0.05 interaction between prenatal treatment and postnatal maternal care. (TIFF) [file pone.0305256.s009.tiff]

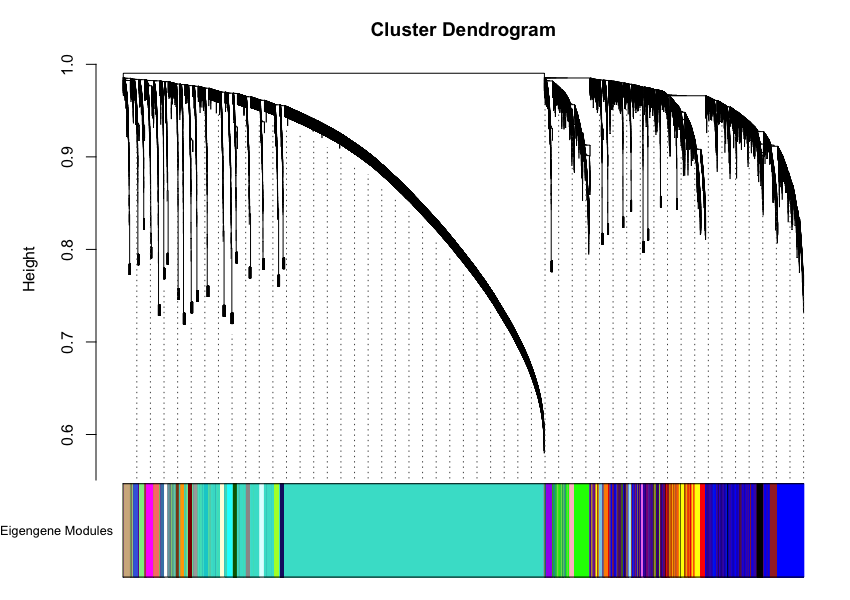

Supplement: S9 Fig — (TIFF) [file pone.0305256.s010.tiff]
